# Distribution of acid-sensing ion channel subunits in human sensory neurons contrasts with that in rodents

**DOI:** 10.1093/braincomms/fcac256

**Published:** 2022-11-02

**Authors:** Melina Papalampropoulou-Tsiridou, Stephanie Shiers, Feng Wang, Antoine G Godin, Theodore J Price, Yves De Koninck

**Affiliations:** CERVO Brain Research Centre, Québec Mental Health Institute, Québec, QC G1J 2G3, Canada; Graduate Program in Neuroscience, Université Laval, Québec, QC G1V 0A6, Canada; Center for Advanced Pain Studies and Department of Neuroscience, University of Texas at Dallas, 800 W Campbell Rd, Richardson, TX 75080, USA; CERVO Brain Research Centre, Québec Mental Health Institute, Québec, QC G1J 2G3, Canada; CERVO Brain Research Centre, Québec Mental Health Institute, Québec, QC G1J 2G3, Canada; Graduate Program in Neuroscience, Université Laval, Québec, QC G1V 0A6, Canada; Department of Psychiatry and Neuroscience, Université Laval, Québec, QC G1V 0A6, Canada; Center for Advanced Pain Studies and Department of Neuroscience, University of Texas at Dallas, 800 W Campbell Rd, Richardson, TX 75080, USA; CERVO Brain Research Centre, Québec Mental Health Institute, Québec, QC G1J 2G3, Canada; Graduate Program in Neuroscience, Université Laval, Québec, QC G1V 0A6, Canada; Department of Psychiatry and Neuroscience, Université Laval, Québec, QC G1V 0A6, Canada

**Keywords:** acid-Sensing ion channels, human dorsal root ganglia, primary sensory neurons, *in situ* hybridization, species differences

## Abstract

Acid-sensing ion channels (ASICs) play a critical role in nociception in human sensory neurons. Four genes (*ASIC1, ASIC2, ASIC3,* and *ASIC4*) encoding multiple subunits through alternative splicing have been identified in humans. Real time-PCR experiments showed strong expression of three subunits *ASIC1*, *ASIC2*, and *ASIC3* in human dorsal root ganglia; however, their detailed expression pattern in different neuronal populations has not been investigated yet. In the current study, using an *in situ* hybridization approach (RNAscope), we examined the presence of *ASIC1*, *ASIC2*, and *ASIC3* mRNA in three subpopulations of human dorsal root ganglia neurons. Our results revealed that *ASIC1* and *ASIC3* were present in the vast majority of dorsal root ganglia neurons, while *ASIC2* was only expressed in less than half of dorsal root ganglia neurons. The distribution pattern of the three *ASIC* subunits was the same across the three populations of dorsal root ganglia neurons examined, including neurons expressing the REarranged during Transfection (RET) receptor tyrosine kinase, calcitonin gene-related peptide, and a subpopulation of nociceptors expressing Transient Receptor Potential Cation Channel Subfamily V Member 1. These results strongly contrast the expression pattern of *Asics* in mice since our previous study demonstrated differential distribution of *Asics* among the various subpopulation of dorsal root ganglia neurons. Given the distinct acid-sensitivity and activity dynamics among different ASIC channels, the expression differences between human and rodents should be taken under consideration when evaluating the translational potential and efficiency of drugs targeting ASICs in rodent studies.

## Teaser

Papalampropoulou-Tsiridou et al. report broad expression of Acid sensing Ion Channel (ASIC) 1 and ASIC3 subunits in human primary sensory neurons. Moreover, the relative distribution of ASICs was constant across neuronal subpopulations, contrasting with expression patterns in rodents. These differences should be taken under consideration when evaluating the translational potential of ASICs studies in rodents.

## Graphical Abstract

**Graphical abstract:**
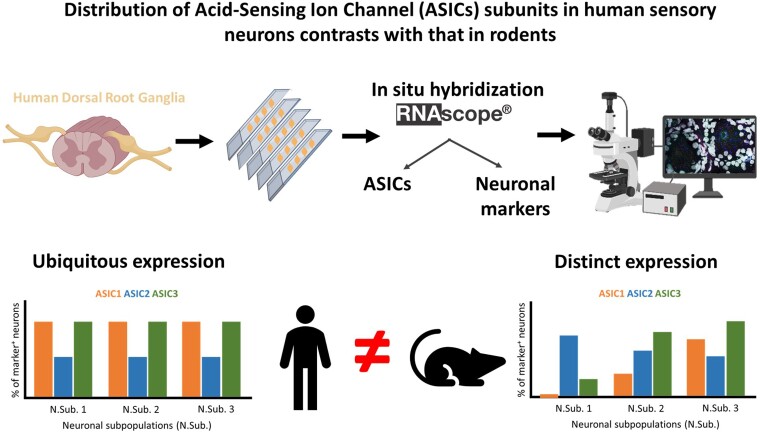

## Introduction

Tissue acidosis is an important signal involved in many normal and pathological conditions. In humans, transient acidosis due to fatiguing exercise,^1^ and enduring acidosis caused by tissue injury, inflammation, and ischemia,^2–4^ could lead to persistent or abnormal pain. Somatosensory afferents are responsible for detecting such noxious acidosis and transmit the signal to the brain.^5^ Proton-sensing ion channels and receptors expressed in somatosensory neurons of human dorsal root ganglion (DRG) include Acid-Sensing Ion Channels (ASICs) and Transient Receptor Potential (TRP) channels,^6,7^ with ASICs being the leading acid-sensing channels in human DRG.^8^ ASICs are members of the degenerin–epithelial sodium channel (DEG–ENaC) family of ion channels^9–11^ with protons being their main ligands.^12^

Four genes (*ASIC1, ASIC2, ASIC3,* and *ASIC4*) encoding at least six subunits (*ASIC1a*, *ASIC1b*, *ASIC2a*, *ASIC2b*, *ASIC3* and *ASIC4*) through alternative splicing, have been discovered in rodents^11^ and humans.^8,13^ Recent RNA-seq analysis of human DRG has shown expression of *ASIC1*, *ASIC2*, and *ASIC3*.^14^ Moreover, ASIC1, ASIC2 and ASIC3 subunits have been detectedin a sub-population of small-diameter sensory neurons in the human DRG by using immunohistochemistry,^15^ however, the molecular properties of ASIC-positive sensory neurons remain unknown. ASICs are involved in many normal and pathological conditions including nociception,^16^ and neuropathic and inflammatory pain^17,18^ respectively. Even though it has been speculated that changes in the function or expression pattern of ASICs might underlie pathological pain syndromes,^19,20^ their expression pattern in neuronal subpopulations within human DRG has not been investigated in detail yet.

In our previous work, we conducted an analysis of the expression pattern of five ASIC subunits, including *Asic1a*, *Asic1b*, *Asic2a*, *Asic2b* and *Asic3* in mouse DRG. Comparing the result of our previous study^19^ with work in rat DRG,^21^ we had concluded that ASICs have a different expression pattern depending on the species.

Considering the important role of ASICs in nociception, and the fact that these channels demonstrate a different expression pattern in DRG depending on the species under investigation, it is a necessity for translational research to explore the distribution of these channels in specific populations of human DRG neurons. This need is further highlighted by many recent studies showing pervasive differences between mouse and human DRG neurons.^22–24^

In the current study, we used an *in situ* hybridization approach (RNAscope) to target ASIC subunits in human DRG and performed quantitative assessment of their expression pattern in different populations of sensory neurons with a focus on nociceptors and markers with high clinical significance. More specifically, we targeted neurons expressing mRNAs for Rearranged during Transfection (*RET*) receptor tyrosine kinase as this receptor is important in signaling axonal outgrowth, cell survival during peripheral nervous system development, and the health and stability of nonpeptidergic nociceptors,^25–28^ Calcitonin gene-related peptide (*CALCA*) due to its high expression in nociceptors,^29,30^ and finally Transient Receptor Potential Cation Channel Subfamily V Member 1 (*TRPV1*) as a marker for human nociceptors that are also activated by extracellular acidification.^30,31^ Our results reveal that the three ASIC subunits, including *ASIC1*, *ASIC2,* and *ASIC3*, show a very different expression pattern from what has been previously observed in rodents.

The differences between species, when it comes to the expression pattern of ASICs, need to be taken under consideration during the interpretation of results from rodent studies and their translational potential to humans.

## Materials and methods

### Human DRG extraction, fixation, and sectioning

All human tissue procurement procedures were approved by the Institutional Review Boards at the University of Texas at Dallas and the Research Ethics Board affiliated with CIUSSS de la Capitale-Nationale. Human lumbar DRGs were procured from organ donors within four hours of cross-clamp and from neurologic determination of death through our collaboration with the Southwest Transplant Alliance. Upon arrival to the research facility, human DRGs from three donors (Table 1) were embedded with OCT in a cryomold by adding small volumes of OCT over dry ice to avoid thawing. All tissues were sectioned using a cryostat at thickness of 20 μm and sections were placed directly onto SuperFrost Plus charged slides (12-550-15, Thermo Fisher Scientific). Sections were only briefly thawed to adhere to the slide but were immediately returned to the −20°C cryostat chamber until completion of sectioning. Slides were removed from the cryostat and immediately fixed with cold (4°C) 10% formalin for 15 min as per Advanced Cell Diagnostics (ACD) guidelines. The tissues were then dehydrated in 50% ethanol (5 min), 70% ethanol (5 min), and 100% ethanol (10 min) at room temperature. The dehydrated sections were then stored in 100% ethanol at −30°C as per the ACD protocols.

**Table 1:** Donor information

	Age	Sex	Cause of death
Donor 1	34	female	opioid overdose
Donor 2	53	male	cardiac arrest
Donor 3	29	male	head trauma

### Tissue quality check

Prior to the employment of the RNAscope technique from ACD for ISH, we validated the procedure and the quality of the RNA using positive and negative control probes provided by the manufacturer. The positive control probes include RNA Polymerase II Subunit A (*POLR2A*), peptidylprolyl isomerase B (*PPIB*), and Ubiquitin C (*UBC*) (320861, ACD), while the negative control probe is dihydrodipicolinate reductase (*DapB*) of *Bacillus subtilis* strain (320871, ACD) and was used to access the non-specific labeling. All the probes were visualized with the RNAscope Fluorescent Multiplex Assay v.1 (Multiplex Assay; 320850, ACD) using human DRG sections. All three samples showed signal for the three positive control housekeeping genes including *POLR2A*, *PPIB*, and *UBC*, with very little variation among samples, and no clear signal was observed for the non-mammalian gene control probe *DapB* (Supplementary Fig. 1). As per the ACD guidelines, sections that have been previously dehydrated and kept in 100% ethanol at −30°C can maintain the RNA well preserved for up to one week. However, we showed that high quality RNA maintains its integrity after four weeks (Supplementary Fig. 2).

### RNAscope assay

The Multiplex Assay was performed based on the guidelines provided by ACD as it has been previously described.^30^ Prior to commencing the ISH, slides were stored in 100% ethanol at −30°C. Before starting the experiments, slides were air dried completely and then boundaries were drawn around the sections using a hydrophobic pen (ImmEdge PAP pen; Vector Labs). When hydrophobic boundaries had dried, protease IV (322340, ACD) was added on each sample until fully covered. The protease IV incubation period was optimized as suggested by ACD. A 15-min protease digestion at room temperature was used for all human DRG experiments. Slides were then washed briefly in 1 × PBS (0.022 M NaH_2_PO_4_, 0.08 M Na_2_HPO_4_, 0.15 M NaCl; pH 7.4) at room temperature. Glass slides were placed in a prewarmed tray and a mixture of RNA probes at Channel one, Channel two, and Channel three (50:1:1 dilution, as per the company’s guidelines) was pipetted on each section until the DRGs were fully covered. The slides were incubated at 40°C for two hours. Then, the ISH signal was revealed using the MultiplexAssay following the manufacturer’s instructions. Briefly, the slides were incubated with Amplifiers 1, 2, 3, and 4 at 40°C for 30, 15, 30, and 15 min, respectively. We used the Amplifier 4—alt C for all the experiments (Channel one = Atto 550, Channel two = Atto 647, Channel three = Alexa 488). Afterwards, human DRG sections were incubated in DAPI staining solution provided by ACD for 45 s before being washed, air dried, and mounted with a Dako Fluorescent mounting medium (S302380-2, Agilent). The probes used for the experiments were: *ASIC1* (576251, ACD), *ASIC2* (576261 and 576261-C2, ACD), *ASIC3* (576271 and 576271-C3, ACD), *CALCA* (605551-C2, ACD), *RET* (424871-C3, ACD), and *TRPV1* (415381-C2, ACD). We performed six independent experiments to reveal the expression pattern of *ASICs*. Three experiments focused on one *ASIC* subunit, *RET*, and *CALCA* (exp. #1: *ASIC1*/*CALCA*/*RET*, exp. #2: *ASIC2*/*CALCA*/*RET*, exp. #3: *ASIC3*/*CALCA*/*RET*). We then conducted a fourth experiment to investigate the co-expression of ASICs (exp. #4: *ASIC1*/*ASIC2*/*ASIC3*). We, finally, investigated the expression of *TRPV1*, *ASIC1*, and *ASIC3* (exp. #5: *ASIC1*/*TRPV1*/*ASIC3*) in one experiment, and the expression of *ASIC2* and *TRPV1* in a different experiment (exp. #6: *ASIC2*/*TRPV1*).

### Confocal imaging

Images were captured using a confocal microscope (Zeiss, LSM700; Oberkochen, Germany) equipped with a 40 × objective using the ZEISS ZEN software. The microscope settings (laser power, gain, and offset) were optimized in individual imaging session and were kept unchanged within the same experiment for a given human sample. Four sections were imaged per human DRG per experiment. A z-stack throughout the whole thickness (20 μm) of the DRG section was acquired at step of 2 μm and the maximum projection of the z-stack images was used for analysis and presented in the results section. The reason this approach was chosen was to avoid false negative neurons. We ensured that there was no overlapping between neurons hence no false co-localization was noticed. Due to the maximum projection of the z-stack images, some signal falsely appears to be inside the surrounding cells, instead of neurons. When the experimenter observed this issue, the original z-stack images were consulted to verify the origin of the signal and confirmed that the signal was indeed inside the neurons (Supplementary Fig. 3).

### Image analysis

Large globular structures and auto-fluorescent signal are present in all three channels (488, 550, and 647), which appears white in the overlay images were considered to be background due to lipofuscin and was not analyzed. The total number of neurons per image was acquired by over-brightening the image to reveal background autofluorescence. All cells that were clearly outlined by DAPI (satellite cell) signal and contained lipofuscin were analysed.^30,32^

To analyze the signal from the images acquired, we used a custom-built MATLAB (MathWorks) code to accelerate the process. This custom-made code was used to count the positive and negative neurons and to measure the neuronal area of positive neurons by drawing polygonal regions of interest around the neurons following the DAPI outline.

A neuron was counted as positive when three or more dots of RNAscope signal were present. We also observed clustering of signal which is common in RNAscope experiments when the expression levels are very high. In addition to the clustering observed in the cytoplasm, in some images, nuclear labeling of RNA targets was noticed. As all mRNAs are transcribed inside the nucleus, this phenomenon occurs regularly when the gene of interest has high level of transcription, which is usually associated with high level of expression.

### Statistical analysis

Graphs were generated and statistical analysis were performed by using GraphPad Prism version 7 (GraphPad Software, Inc. San Diego, CA USA). All results in the graphs are presented as mean ± standard error of the mean (SEM) and the three samples are shown as individual points. Four sections per sample and approximately one hundred neurons per section were analysed and averaged. Welch One-way ANOVA was used to compare the size distribution between different populations of gene(s) positive neurons, with *P* < 0.05 considered significant. The results of this statistical analysis are provided in the relevant results section and in the figure legends for each experiment. For the pie charts, the total number of ASIC positive neurons or total number of neurons analyzed can be found in the figure legend.

### Data availability statement

The data that support the findings of this study are available from the corresponding author, upon reasonable request.

## Results

### *ASIC1* expression in *CALCA* and *RET* positive neurons

**Figure 1:**
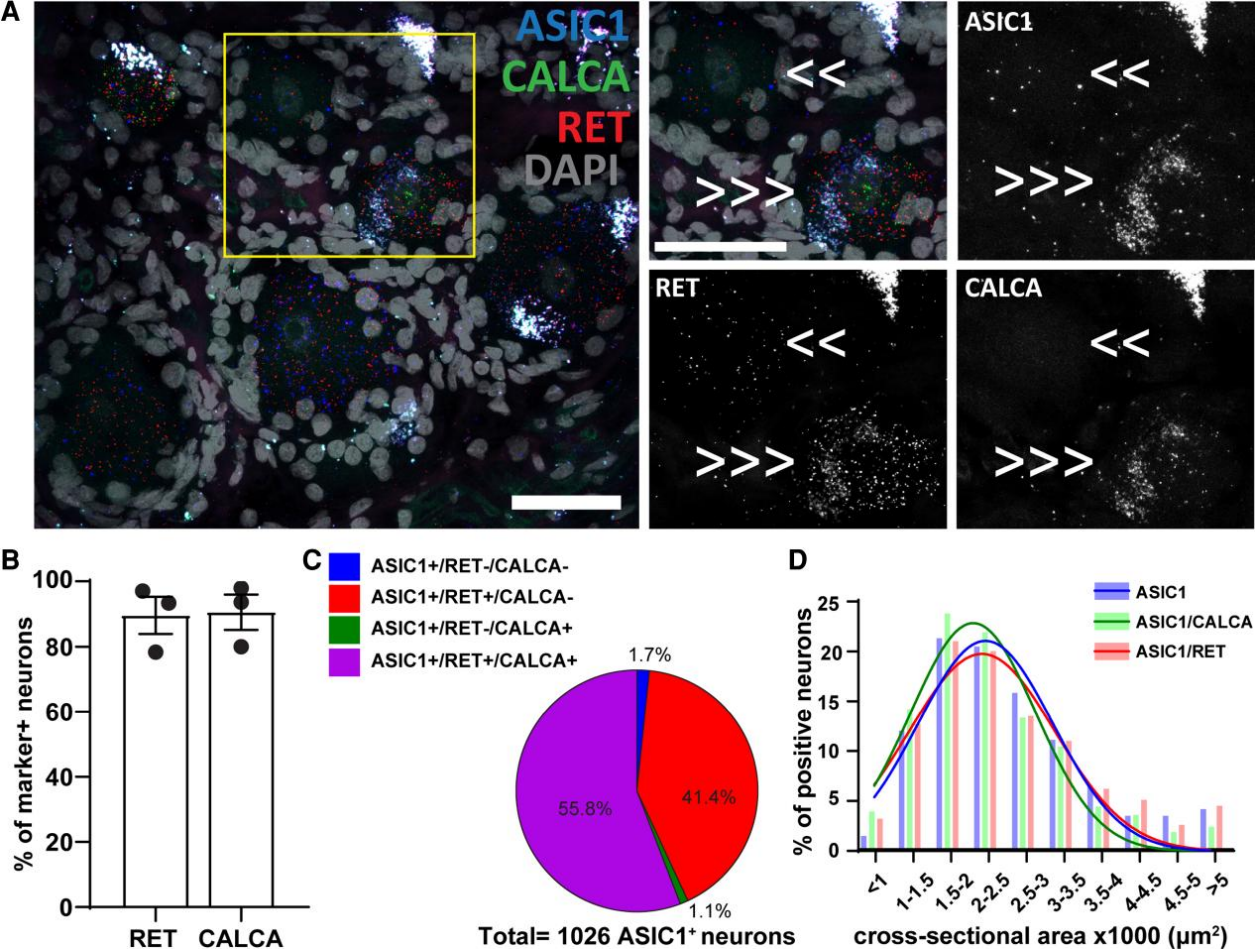
***ASIC1* expression in *CALCA*^+^and *RET*^+^ primary sensory neurons.** (**A**) Representative confocal image showing the expression of *ASIC1* (Acid-Sensing Ion Channel 1) in primary sensory neurons expressing *RET* (receptor tyrosine kinase RET) and *CALCA* (Calcitonin gene-related peptide). Τhe region within the rectangle is further highlighted on the right-side images. Double arrows point to cells which are double positive of *ASIC1* and *RET*, and triple arrows point to cells expressing all the three genes. Scale bar = 50 μm. (**B**) Percentage of marker positive neurons expressing *ASIC1*; *n* = 3 DRGs from three donors. (**C**) Pie chart showing the distribution of *ASIC1* positive neurons in the experiment #1; *n* = 1026 *ASIC1*^+^ neurons from three samples. (**D**) Histogram along with fitting curves of the neuronal size distribution (bin = 500 μm^2^) of *ASIC1*^+^ neurons and *ASIC1*/marker double positive neurons in human DRGs; Welch one-way ANOVA, W(DFn, DFd) = 2.112(2.000,3.874), *P* = 0.24, 584–1026 neurons from three samples. Detailed sample size can be found in Supplementary Table 1

**Figure 2:**
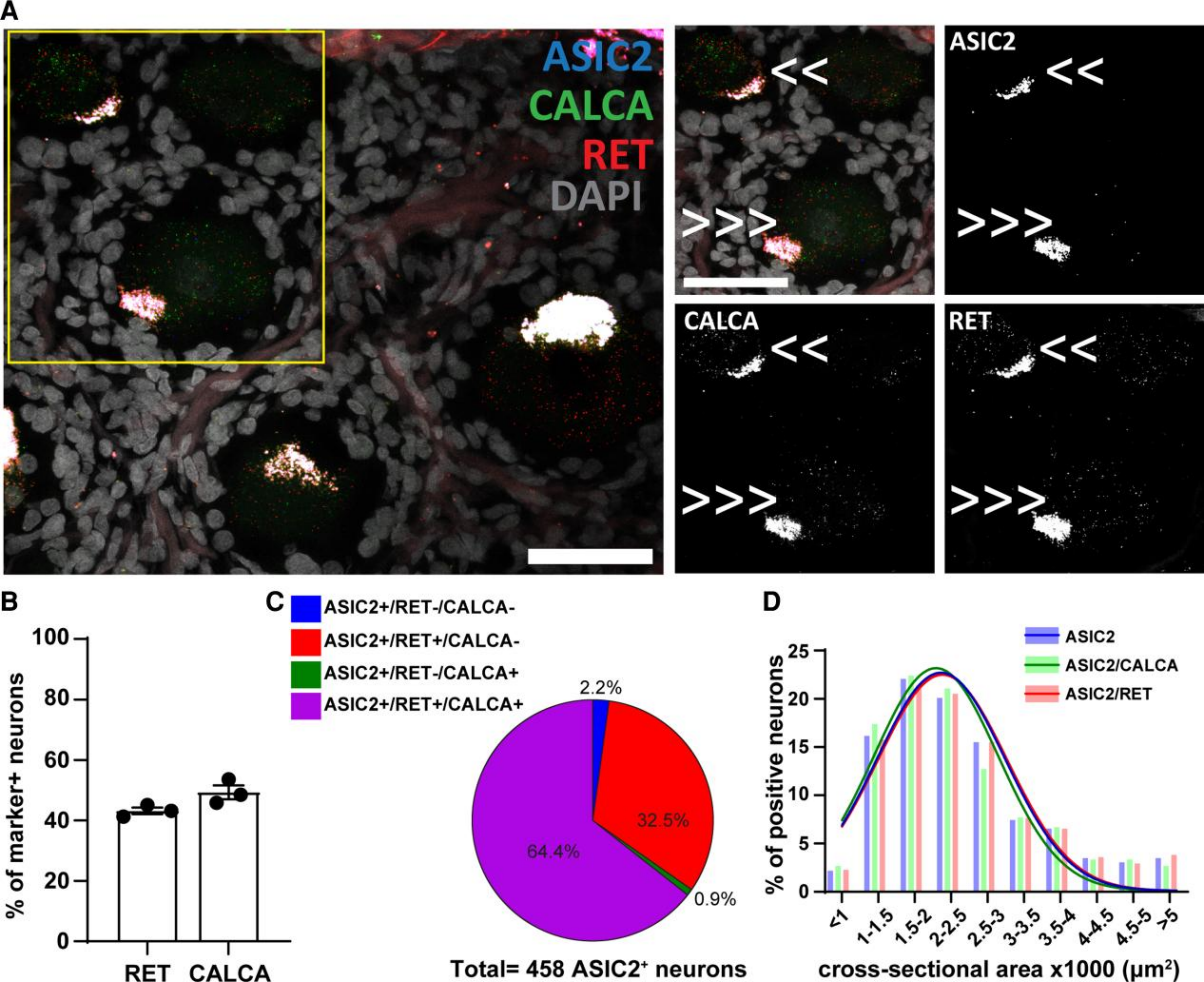
***ASIC2* expression in *CALCA*^+^and *RET*^+^ primary sensory neurons.** (**A**) Representative confocal image showing the expression of *ASIC2* (Acid-Sensing Ion Channel 2) in primary sensory neurons expressing *RET* (receptor tyrosine kinase RET) and *CALCA* (Calcitonin gene-related peptide). Τhe region within the rectangle is further highlighted on the right-side images. Double arrows point to cells expressing two genes, and triple arrows point to cells expressing all the three genes. Scale bar = 50 μm. (**B**) Percentage of marker positive neurons expressing *ASIC2*; *n* = 3 DRGs from three donors. (**C**) Pie chart showing the distribution of *ASIC2* positive neurons in the experiment #2; *n* = 458 *ASIC2*^+^ neurons from three samples. (**D**) Histogram along with fitting curves of the neuronal size distribution (bin = 500 μm^2^) of *ASIC2*^+^ neurons and *ASIC2*/marker double positive neurons in human DRGs; Welch one-way ANOVA, W(DFn, DFd) = 0.4565(2.000,4.000) *P* = 0.66, 299–458 neurons from three samples. Detailed sample size can be found in Supplementary Table 1

**Figure 3:**
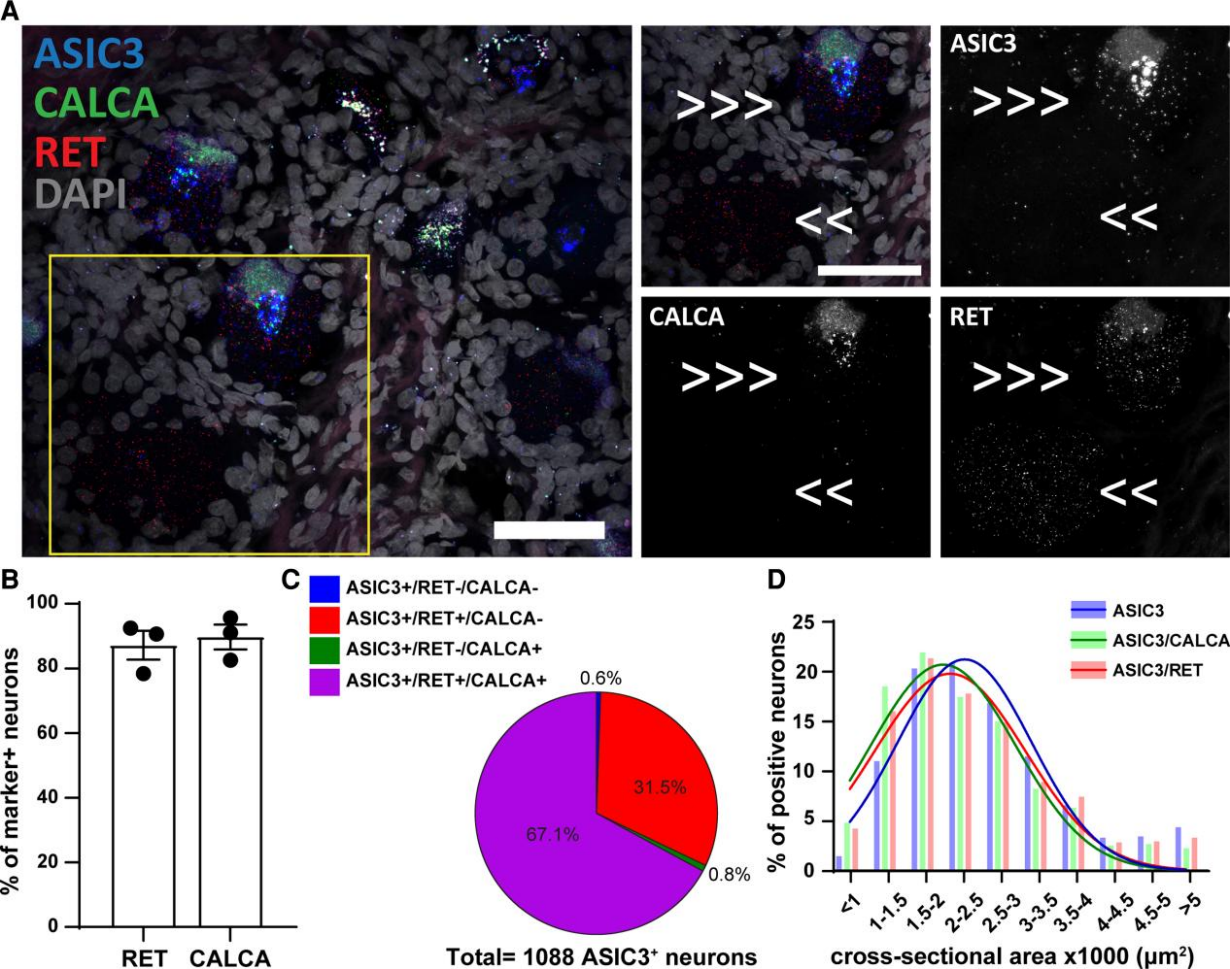
***ASIC3* expression in *CALCA*^+^ and *RET*^+^ primary sensory neurons.** (**A**) Representative confocal image showing the expression of *ASIC3* (Acid-Sensing Ion Channel 3) in primary sensory neurons expressing *RET* (receptor tyrosine kinase RET) and *CALCA* (Calcitonin gene-related peptide). Τhe region within the rectangle is further highlighted on the right-side images. Double arrows point to cell expressing *ASIC3* and *RET*, and triple arrows point to cells expressing all the three genes. Scale bar = 50 μm. (**B**) Percentage of marker positive neurons expressing *ASIC3*; *n* = 3 DRGs from three donors. (**C**) Pie chart showing the distribution of *ASIC3* positive neurons in the experiment #3; *n* = 1088 *ASIC3*^+^ neurons from three samples. (**D**) Histogram along with fitting curves of the neuronal size distribution (bin = 500 μm^2^) of *ASIC3*^+^ neurons and *ASIC3*/marker double positive neurons in human DRGs; Welch one-way ANOVA, W(DFn, DFd) = 4.950(2.000,3.948), *P* = 0.08, 739–1088 neurons from three samples. Detailed sample size can be found in Supplementary Table 1

We sought to investigate the expression of the three ASIC subunits in human DRG neuronal subpopulations expressing *RET* and *CALCA*. In post-mortem human DRGs, we observed a wide expression of *RET* (Figs 1A, 2A and 3A) in almost all (93%) primary sensory neurons. Similar results with the high occurrence of *RET* mRNA in sensory neurons has been reported in previous studies.^33^  *CALCA* was expressed in 57% of all DRG neurons, which is consistent with previous results from human DRGs using RNAscope.^30^

Our RNAscope experiment showed that *ASIC1* is present in almost all human primary sensory neurons, which resulted in about 90% of *RET^+^* and *CALCA*^+^ neurons expressing *ASIC1* (Fig. 1A and B). In the neurons expressing *ASIC1*, more than half of them express both *RET* and *CALCA* (56%) while 41% express only *RET* but not *CALCA* (Fig. 1C).

We proceeded to measure the cross-neuronal size of *ASIC1*^+^ neurons, and we did not observe any statistically significant differences (Welch one-way ANOVA, W(DFn, DFd) = 2.112(2.000,3.874), *P* = 0.24) in the neuronal size distribution among the whole population and different sub-populations of *ASIC1*^+^ neurons (Fig. 1D).

### *ASIC2* expression in *CALCA* and *RET* positive neurons

We continued investigating the expression of *ASIC2* in *CALCA*^+^ and *RET^+^* neurons. *ASIC2* shows a different pattern compared to *ASIC1*. *ASIC2* is present in 43% of *RET*^+^ and 49% of *CALCA*^+^ neurons (Fig. 2A and B). From the neurons expressing *ASIC2*, the majority express both *RET* and *CALCA* (64%), while 33% express only *RET* but not *CALCA* (Fig. 2C).

Similar to *ASIC1*, we did not observe any differences in the size distribution of the whole population of *ASIC2*^+^ neurons and its different subpopulations (Welch one-way ANOVA, W(DFn, DFd) = 0.4565(2.000,4000) *P* = 0.66) (Fig. 2D).

### *ASIC3* expression in *CALCA* and *RET* positive neurons

The expression pattern of *ASIC3* shares some similarities with the expression pattern of *ASIC1* since this subunit is also expressed in most human primary sensory neurons. More than 85% of *RET*^+^, and almost 90% of *CALCA*^+^ neurons express *ASIC3* (Fig. 3A and B). Similar to the *ASIC1* expression pattern, most of *ASIC3* positive neurons express both *CALCA* and *RET* (Fig. 3C). Like *ASIC1* and *ASIC2*, there were no changes in the size distribution of *ASIC3*/marker-double positive neurons depending on the population (Welch one-way ANOVA, W(DFn, DFd) = 4.950(2.000,3.948), *P* = 0.08)(Fig. 3D).

### Co-expression of *ASIC*s subunits in primary sensory neurons

**Figure 4:**
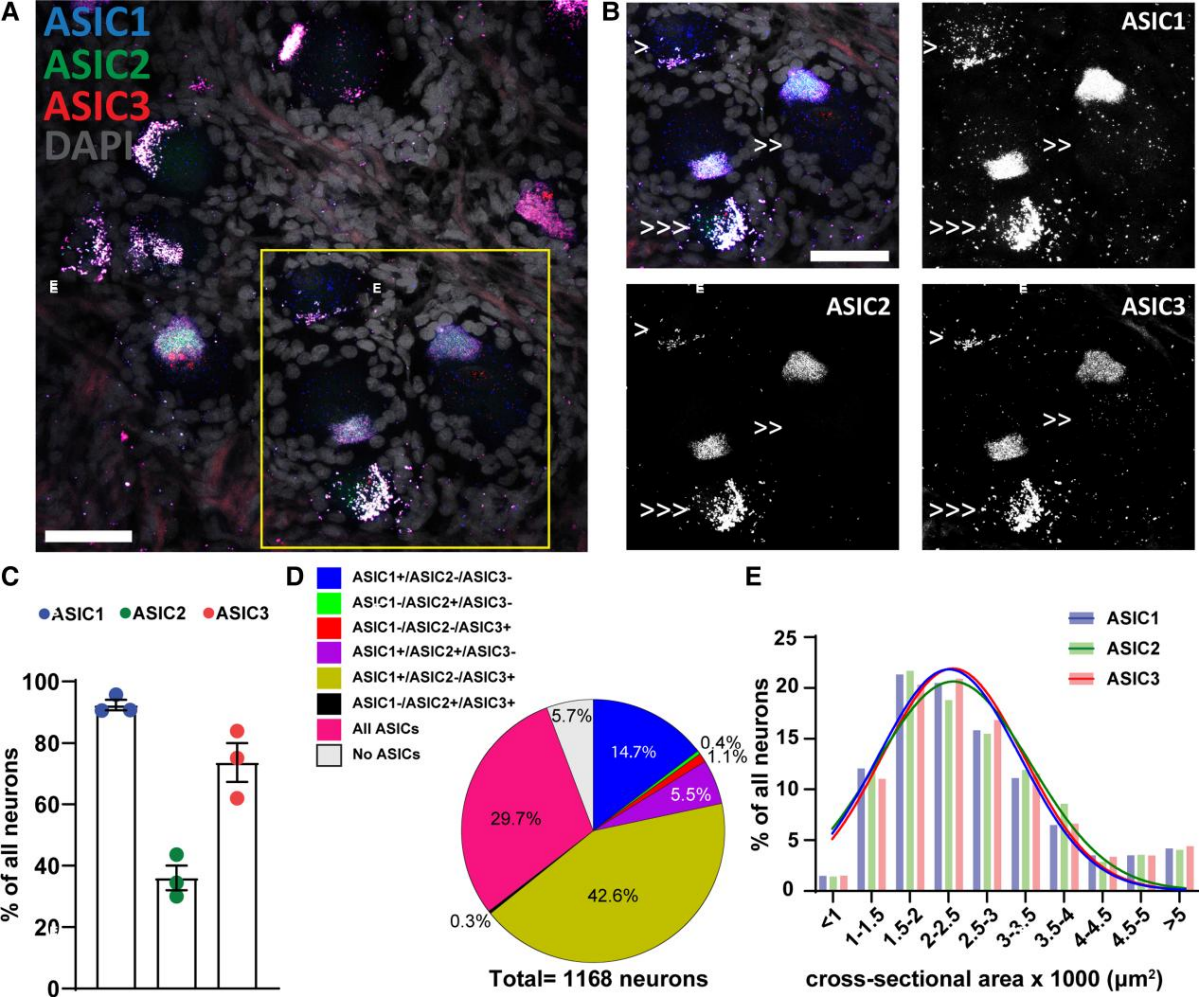
***ASIC*s expression in human primary sensory neurons.** (**A**) Representative confocal image showing the expression of *ASIC1* (Acid-Sensing Ion Channel 1), *ASIC2* (Acid-Sensing Ion Channel 2) and *ASIC3* (Acid-Sensing Ion Channel 3) in primary sensory neurons. (**B**) Τhe region within the rectangle in A is highlighted. Single arrow points to cell expressing only one *ASIC* subunit. Note the neuron pointed with single arrow has strong auto-fluorescent lipofuscin signal, which appears in all three channels. Double arrows point to cells expressing two *ASIC* subunits, and triple arrows point to cells expressing all three *ASIC* subunits. Scale bar = 50 μm. (**C**) Percentage of all DRG neurons expressing different *ASIC* subunits; *n* = 3 DRGs from 3 donors. (**D**) Pie chart showing the distribution of DRG neurons expressing different combinations of *ASIC1*, *ASIC2*, and *ASIC3* from experiment #4; *n* = 1168 neurons from three samples. (**E**) Histogram along with fitting curves of the neuronal size distribution (bin = 500 μm^2^) of *ASIC* positive neurons in human DRGs; Welch one-way ANOVA, W(DFn, DFd) = 0.4797(2.000,3.942), *P* = 0.65, 420–1079 neurons from three samples. Detailed sample size can be found in Supplementary Table 1

We then proceeded to investigate the co-expression of *ASIC1*, *ASIC2,* and *ASIC3* subunits in human primary sensory neurons. Overall, we observed a very different co-expression pattern comparing to our previous observations in mouse sensory neurons. *ASIC1* is expressed in 92% of all sensory neurons express, while *ASIC2* and *ASIC3* are present in about 36 and 74% of all sensory neurons, respectively (Fig. 4A–C). Almost 15% of all human sensory neurons express only *ASIC1* subunit; however, less than 0.5% and 1.5% of all neurons only express *ASIC2* and *ASIC3* subunits, respectively (Fig. 4D). Similar to mouse primary sensory neurons, most human DRG neurons also express two or three different *ASICs* subunits. However, it is worth noting that the most dominant subunit in human DRG is *ASIC1* and not *ASIC3*.^19^ More specifically, *ASIC1*/*ASIC2* double positive but *ASIC3* negative neurons count for 6% of all neurons, while 43% of all neurons are *ASIC1*/*ASIC3* double positive but *ASIC2* negative (Fig. 4D). There is only a very small fraction of neurons (0.3%) that expresses only *ASIC2* and *ASIC3* but not *ASIC1* (Fig. 4D). Finally, all subunits are present in almost 30% of all neurons, while only 6% does not express any *ASIC* subunit (Fig. 4D).

We also analysed the size distribution of *ASIC*^+^ neurons. Interestingly, we did not observe any differences among the neurons expressing different *ASIC* subunits (Welch one-way ANOVA, W(DFn, DFd) = 0.4797(2.000,3.942), *P* = 0.65), which is also different from *Asic*^+^ neurons in mice. The neuronal size of majority of *ASIC*^+^ human DRG neurons were between 1500 μm2 and 2500 μm2 (Fig. 4E).

### Expression of *ASIC*s in *TRPV1* positive neurons

TRPV1 is a marker of a subpopulation of nociceptors in rodents^34,35^ and all nociceptors in humans^30^ and, as ASICs, TRPV1 channels can be activated by extracellular acidification,^31,36,37^ although with lower sensitivity. Thus, it is important to examine colocalization between TRPV1 and ASIC subunits.

**Figure 5:**
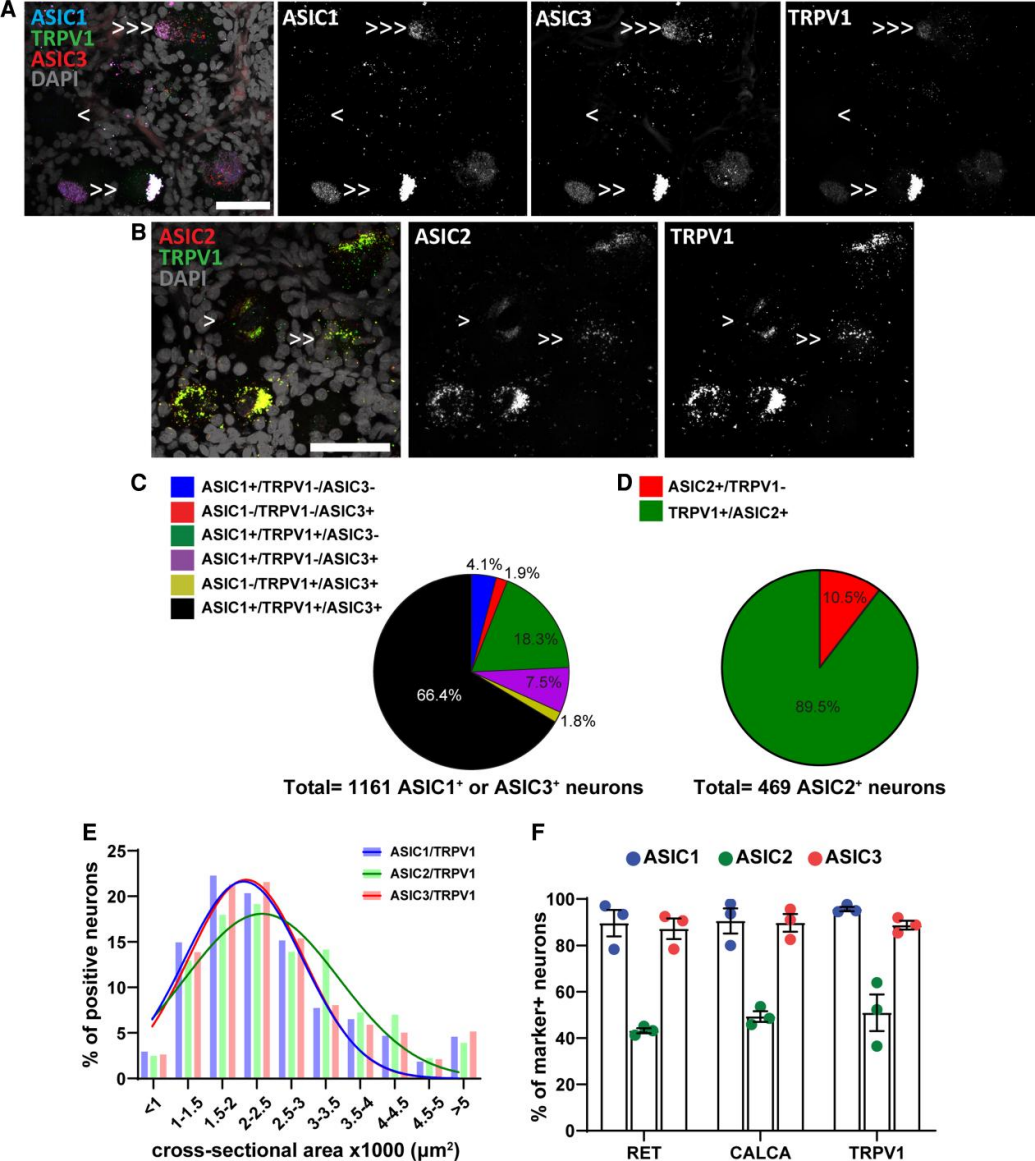
***ASIC*s expression in *TRPV1*^+^ primary sensory neurons.** (**A**) Representative confocal image showing the expression of *ASIC1* (Acid-Sensing Ion Channel 1), *ASIC3*(Acid-Sensing Ion Channel 3) and *TRPV1*^+^ (Transient Receptor Potential Cation Channel Subfamily V Member) sensory neurons. (**B**) Representative confocal image showing the expression of *ASIC2* (Acid-Sensing Ion Channel 2) and *TRPV1*^+^ sensory neurons. Single arrow points to cell expressing only one gene, double arrows point to cells ­­­­­­expressing two genes, and triple arrows point to cells expressing three genes. Scale bar = 50 μm. (**C**) Pie chart showing the distribution of DRG neurons expressing different combinations of *ASIC1*, *ASIC3*, and *TRPV1* from experiment #5; *n* = 1161 *ASIC1* or *ASIC3* positive neurons from three samples. (**D**) Pie chart showing the distribution of DRG neurons expressing different combinations of *ASIC2* and *TRPV1* from experiment #6; *n* = 469 *ASIC2* positive neurons from three samples. (**E**) Histogram along with fitting curves of the neuronal size distribution (bin = 500 μm^2^) of *ASIC*s/*TRPV1* double positive neurons in human DRGs; Welch one-way ANOVA, W(DFn, DFd) = 3.245(2.000, 3.943), *P* = 0.15, 420–983 neurons from three samples. (F) Percentage of marker positive neurons expressing different *ASIC* subunits; *n* = 3 DRGs from three donors. Part of this graph is duplication from Figs 1B, 2B, and 3B. Detailed sample size can be found in Supplementary Table 1

With *in situ* hybridization, we observed that more than 70% of human primary sensory neurons express *TRPV1* (Fig. 5A and B), which is in accordance with a recent study.^30^  *ASIC1*, *ASIC2,* and *ASIC3* are expressed in 95, 51, and 75% of *TRPV1* positive neurons, respectively (Fig. 5F). The experiment investigating the expression of *ASIC1* and *ASIC3* in *TRPV1* neurons revealed that 66% of *ASIC1* or *ASIC3* positive neurons express both subunits and *TRPV1*, only 2% express *ASIC3* and *TRPV1* but not *ASIC1* and 18% express *ASIC1* and *TRPV1* but not *ASIC3* (Fig. 5C).

The experiment investigating the co-expression of *ASIC2* and *TRPV1* showed that *TRPV1* is expressed in almost 90% of neurons expressing *ASIC2* (Fig. 5D). Finally, the size distribution of *ASICs*/*TRPV1* double positive neurons does not change depending on the ASIC subunit (Welch one-way ANOVA, W(DFn, DFd) = 3.245(2.000,3.943), *P* = 0.15) (Fig. 5E).

The overall expression pattern of the three *ASIC* subunits in the three studied populations is presented in Fig. 5F to summarize our results. The distribution pattern of the three *ASIC* subunits (*ASIC1*, *ASIC2*, and *ASIC3*) was the same across the three sub-populations of dorsal root ganglia neurons examined.

## Discussion

In the current study, we investigated the expression of *ASIC1*, *ASIC2,* and *ASIC3* in three subpopulations of primary sensory neurons within human DRGs. Considering the fundamental role of ASIC subunits in several somatosensory processes^38^ and more specifically in tissue acidosis associated pain,^39^ we focused on subpopulations of nociceptors. More specifically, we targeted neurons expressing *RET*, *CALCA*, and *TRPV1*. We demonstrated that *ASIC* subunits expressed in adult human DRG show a differential expression pattern comparing to our previous observations in adult mouse DRG^19^ and reported distribution in rat DRG.^40,41^

The investigation of the co-expression of *ASICs* in human primary sensory neurons and the subsequent comparison of the results with our previous observations in mouse sensory neurons reveal several between-species variations. Based on our previous work, 35%, 50%, and 60% of all DRG neurons expressed *Asic1a*, *Asic2b*, and *Asic3,* respectively, in mouse DRG.^19^ Moreover, heterotrimeric channels appear to be the leading form of ASIC channels with *Asic3* being the most dominant subunit.^19^ In the current study, we showed that *ASIC1*, *ASIC2,* and *ASIC3* are present in 90, 35, and 70% of primary sensory neurons, respectively (Fig. 4C), and, similarly to mouse primary sensory neurons, most of human DRG neurons express two or three subunits, however the most dominant subunit is *ASIC1* and not *ASIC3* (Fig. 1D), which is consistent with the recent study of human sensory neurons’ molecular signatures by using spatial single-cell transcriptomics.^42^

The second striking difference between human and mouse DRG was that the size distribution of *ASIC*^+^ neurons does not change depending on the subunit expressed since *ASIC1*, *ASIC2,* and *ASIC3* showed the same distribution pattern (Fig. 4E), while in mouse DRG, we observed a different distribution pattern depending on the ASIC subunit in the NF200 population.^19^

Our observations related with the *ASICs* expression pattern in different populations surprised us since we expected to see a certain level of variation on the pattern depending on the subpopulation under investigation. Contrary to our initial hypothesis of a differential expression pattern, the percentage of marker positive neurons expressing one of the *ASIC* subunits was the same in the three studied populations with *ASIC1* and *ASIC3* being expressed in most marker positive neurons (*RET*^+^, *CALCA*^+^, and *TRPV1*^+^) and *ASIC2* being expressed in between 40 and 50% of marker positive neurons (Fig. 5F). On the same note, there is no difference in the size distribution of marker/*ASIC* double positive neurons depending on the population and the subunit under investigation.

It is important to note that, in human DRGs, the distinction between peptidergic and non-peptidergic subpopulations is not straight-forward as in rodents. Commonly used markers, for example CGRP (*CALCA*) and RET or P2X3, which in rodents can well separate these two subpopulations, show significant levels of overlapping in human. Up to date, a marker targeting specifically one of these two subpopulations in humans has not been identified yet. We opted to focus on *CALCA*, *RET,* and *TRPV1* as they are widely used markers in human research and especially *TRPV1* targets a subpopulation of nociceptors in human DRGs.

The three samples used in the current study gave very consistent results with little variation (Fig. 1B, 2B, 3B, 4C, 5F) regardless of the sex, age, and cause of death. Even though one study reported sex differences in the expression of ASICs in mice DRGs innervating the urinary bladder^43^ interestingly, a recent paper using spatial single-cell transcriptomics did not reveal sex differences in *ASICs* in human DRGs.^42^

Overall, we observed a very different expression pattern compared to our previous study and work conducted in rat DRGs^40,41^ when it comes to the expression pattern of *ASICs*. These differences are consequence of between-species variations related with the expression of *ASICs* per se that have been demonstrated in previous work,^19,44^ and with the phenotypic differences of DRG neurons as well. These discrepancies need to be taken under consideration when evaluating the translational potential of rodent studies.

Species differences regarding the expression and function of ASICs have been suggested to be an environmental adaptation. For instance, in most rodents, ASIC3 subunit can form homotrimeric channel whose activation leads to a biphasic current with a fast inactivating and a slow sustained phase.^45,46^ In contrast, in naked-mole rats, ASIC3 does not form functional homotrimeric channels.^47^ This functional difference in ASIC3 might reflect an adaptation of the naked mole-rat to the high-carbon dioxide levels in their habitat.^47^ On the other hand, ASIC3a, which is the most dominant ASIC3 variant in human DRG, has been shown to be a sensor for both extracellular acidification and alkalization,^48^ while in rodents, ASIC3 is only sensitive to acidification, but not alkalization.^49^

Considering the variable roles of different ASIC subunits in neuronal physiology and behavior, it is important for translational research, to better understand ASICs expression and function in the human nervous system since these two attributes could alter neuronal acid sensitivity. Our current results show that *ASICs* expression does not vary among different subpopulations, which could further imply that the response to extracellular acidification is universal between several subtypes of sensory neurons. Future functional studies should be conducted to further support this hypothesis. Finally, preclinical research studies have reported a connection between ASICs and two well-known pain treatments: Non-Steroidal Anti-Inflammatory Drugs (NSAIDs)^21,50^ and opioids.^17^ In addition, amiloride, which is a generic ASICs blocker, is already used as medication for management and treatment of hypertension and heart failure.^51^ Meanwhile, amiloride has demonstrated promising clinical efficacy in migraine patients from a small open-labelled pilot study.^52,53^ Future work should focus on the potential analgesic effects of amiloride and further investigate the mechanisms underlying the effects of NSAIDs and opioids on ASICs in humans. An important step towards a more precise interpretation of results from rodents and their translational potential to humans is to take under consideration differences between species when it comes to the expression pattern of ASICs.

## Supplementary Material

fcac256_Supplementary_Data

## Data Availability

The data that support the findings of this study are available from the corresponding author, upon reasonable request.

## Abbreviations

ASIC(s) =: Acid-Sensing Ion Channel(s)
CALCA =: Calcitonin gene-related peptide
DAPI =: 4’,6-diamidino-2-phenylindole
DRG =: Dorsal Root Ganglia
DEG–ENaC =: degenerin–epithelial sodium channel
ISH =: *in situ* hybridization
RET =: REarranged during Transfection receptor tyrosine kinase
TRPV1 =: Transient Receptor Potential Cation Channel Subfamily V Member 1
