# Supplementary material for: Distribution of acid-sensing ion channel subunits in human sensory neurons contrasts with that in rodents
Source: Brain Commun. 2022 Nov 2;4(6):fcac256. doi: 10.1093/braincomms/fcac256 (PMC9629378; doi:10.1093/braincomms/fcac256)
Supplement: fcac256_Supplementary_Data [file fcac256_supplementary_data.pdf]

Supplementary figures

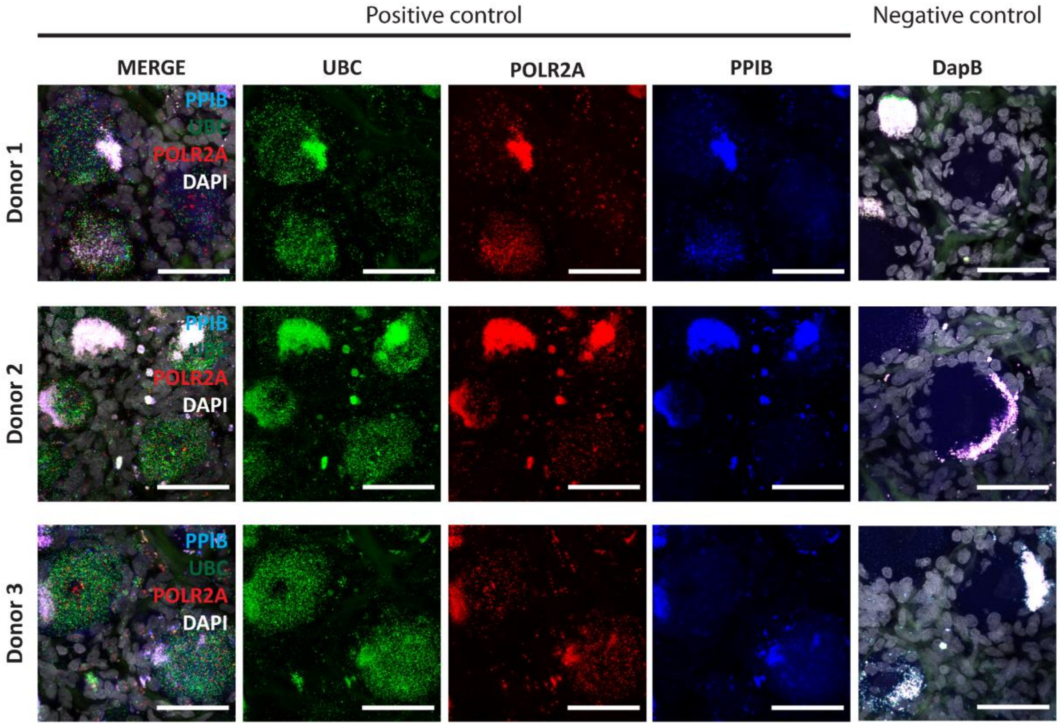

Supplementary figure 1: Validation of the RNAscope approach.
The signal levels of positive control probes targeting eukaryotic housekeeping genes *POLR2A* (RNA Polymerase II Subunit A), *PPIB* (peptidylprolyl isomerase B), and *UBC* (Ubiquitin C), were consistent with our expectations. More specifically, *UBC* was expected to have the highest expression, *POLR2A* has moderate to low expression levels, and *PPIB* has moderate to high expression levels. The negative control probe (*DapB* - dihydrodipicolinate reductase of Bacillus subtilis strain) did not show any signal. For all experiments, human DRG sections from three donors were used. Scale bar = 50 μm.

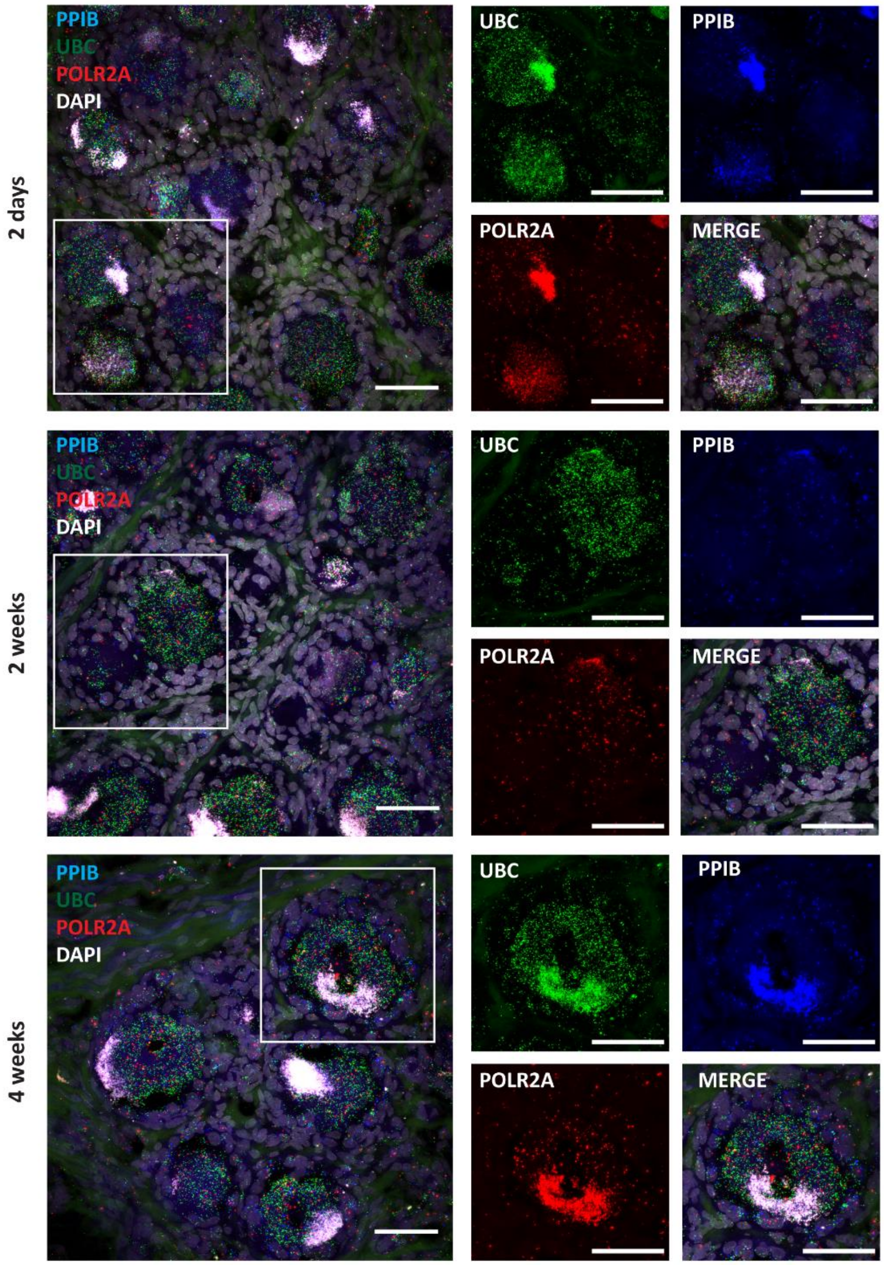

Supplementary figure 2: Validation of the RNA quality after a month of storage in 100% ethanol at -30°C.

Representative images of human DRG sections two days, two weeks, and four weeks after sequential dehydration and storage in 100% ethanol at -30°C. The region within the white square is further highlighted on the right-side images. The signal is from control probes targeting eukaryotic housekeeping genes *POLR2A (*RNA Polymerase II Subunit A), *PPIB (*peptidylprolyl isomerase B), and *UBC (*Ubiquitin C). Scale bar = 50 µm

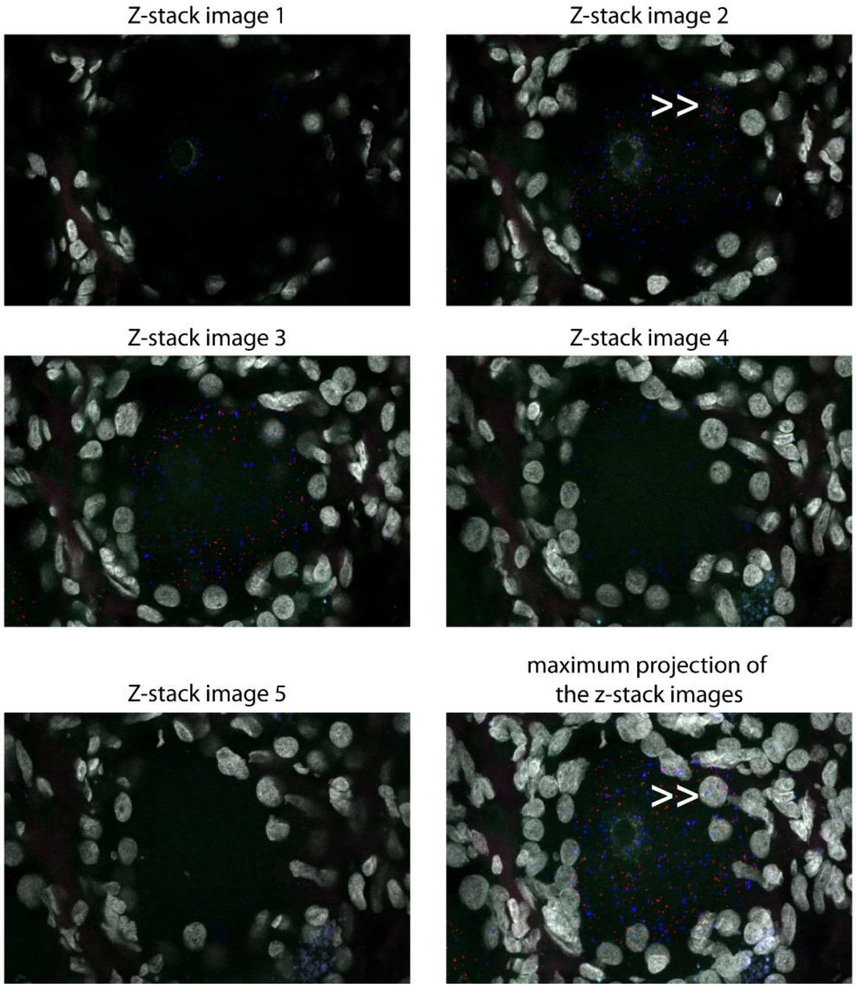

Supplementary figure 3: Example of stack images and final maximum projection image. Representative example of RNAscope signal (Acid-Sensing Ion Channel 1 in blue and receptor tyrosine kinase RET in red). This image is a zoomed-in image from Figure A1. The double arrow points towards RNAscope signal that is located inside the neuron in Z-stack image 2 and not in the satellite cells as it appears in the final maximum projection image.

Supplementary table 1

Below we are providing the number of neurons analysed per donor for each one of the five experiments conducted for this paper and presented in the Figures.

	Figure 1C	Figure 1D		
	ASIC1$^+$	ASIC1$^+$	ASIC1/CALCA	ASIC1/RET
Donor 1	368	368	242	358
Donor 2	288	288	120	275
Donor 3	370	370	222	365
Total	1026	1026	584	998

	Figure 2C	Figure 2D		
	ASIC2$^+$	ASIC2$^+$	ASIC2/CALCA	ASIC2/RET
Donor 1	140	140	115	136
Donor 2	164	164	74	157
Donor 3	154	154	110	151
Total	458	458	299	444

	Figure 3C	Figure 3D		
	ASIC3$^+$	ASIC3$^+$	ASIC3/CALCA	ASIC3/RET
Donor 1	408	408	295	397
Donor 2	346	346	269	344
Donor 3	334	334	175	332
Total	1088	1088	739	1073

	Figure 4D	Figure 4E		
	total number	ASIC1	ASIC2	ASIC3
Donor 1	400	383	120	300
Donor 2	378	343	130	234
Donor 3	390	353	170	327
Total	1168	1079	420	861

	Figure 5C	Figure 5D	Figure 5E		
	ASIC1$^+$ or ASIC3$^+$	ASIC2$^+$	ASIC1/TRPV1	ASIC2/TRPV1	ASIC3/TRPV1
Donor 1	356	118	294	106	214
Donor 2	422	165	368	138	274
Donor 3	383	186	321	176	304
Total	1161	469	983	420	792
